# YBX1 is required for assembly of viral replication complexes of chikungunya virus and replication of multiple alphaviruses

**DOI:** 10.1128/jvi.02015-24

**Published:** 2024-12-31

**Authors:** Zhen-Qi Li, Li-Xin Zhao, Su-Yun Wang, Chu-Yu Hu, Yan-Yi Wang, Yan Yang

**Affiliations:** 1 Key Laboratory of Virology and Biosafety, Wuhan Institute of Virology, Chinese Academy of Sciences 74614 , Wuhan, Hubei, China; 2 University of Chinese Academy of Sciences 74519 , Beijing, China; 3 School of Life Sciences, Division of Life Sciences and Medicine, University of Science and Technology of China 12652 , Hefei, Anhui, China; 4 State Key Laboratory of Virology, Wuhan Institute of Virology, Chinese Academy of Sciences 74614 , Wuhan, Hubei, China; Loyola University Chicago - Health Sciences Campus, Maywood, Illinois, USA

**Keywords:** alphaviruses, cellular factor, YBX1, viral replication

## Abstract

Chikungunya virus (CHIKV), an enveloped positive-sense RNA virus, is a member of the alphaviruses and cause fever and arthralgia in humans. We performed genome-wide CRISPR/Cas9-based screens and identified Y-box binding protein 1 (YBX1) as an essential cellular factor for CHIKV. Deficiency of YBX1 inhibited CHIKV RNA replication and impaired virus production. Upon CHIKV infection, YBX1 showed a striking re-localization to viral replication complexes (vRCs), where it co-localized with CHIKV nsP3 and dsRNA intermediates. YBX1 directly interacted with CHIKV nsP3, and mutation of the YBX1-binding motif in CHIKV nsP3 suppressed viral replication in host cells. Furthermore, YBX1 bound to viral RNA and increased the viral RNA-binding activity of CHIKV nsP3. Consistently, the RNA-binding activity of YBX1, as well as the ability of nsP3 to bind to YBX1, was required for efficient CHIKV replication. In addition to CHIKV, YBX1 was also essential for replication of all examined alphaviruses including the prototypic alphavirus. Our findings suggest that YBX1 acts as a scaffold for assembly of chikungunya vRCs and an important factor for replication of multiple alphaviruses, which may serve as a potential target for the development of anti-alphavirus therapies.

**IMPORTANCE:**

Alphaviruses are a group of mosquito-transmitted, enveloped, positive-strand RNA viruses in the *Togaviridae* family. Most alphaviruses are important pathogens that continue to cause human disease ranging from severe and potentially fatal neurological disease to chronic arthritic disease on a global scale. Here, we found that YBX1 promotes binding of CHIKV genomic RNA to nsP3, which is a key component of the replication complex, and is therefore pivotal for CHIKV replication. Deficiency of YBX1 results in reduced replication of multiple alphaviruses, including arthritogenic and encephalitic alphaviruses. These findings suggest that YBX1 is an important cellular factor for multiple alphaviruses and a potential target for preventing alphavirus infections.

## INTRODUCTION

Alphaviruses are enveloped positive-strand RNA viruses of the *Togaviridae*, which are mostly transmitted to vertebrates by mosquitos and cause human diseases (1–3). According to clinical symptoms caused by the viruses and their geographical distribution, alphaviruses can be divided into old-world alphaviruses (OWs) and new-world alphaviruses (NWs). The OWs include chikungunya virus (CHIKV), Sindbis virus (SINV), Semliki Forest virus (SFV), O’nyong-nyong virus (ONNV), Mayaro virus, and Ross River virus. Human infections with OWs are typically characterized by fever, rash, as well as arthralgia and myalgia, which can persist for months to years after infection (4–6). On the other hand, human infections with NWs, such as Eastern, Western, and Venezuelan equine encephalitis viruses (VEEV), can cause severe neurological impairment and death (7). Among them, CHIKV is the most widespread alphavirus which causes recurrent outbreaks of chikungunya fever globally (8, 9).

Alphaviruses are structurally similar and share a common life cycle (10). Mature particles are approximately 70 nm in diameter and comprise a nucleocapsid enveloped by a host-derived lipid bilayer membrane (11). The genome (G RNA) of alphavirus is about 11–12 kb in length with a 5′ cap0 structure and a 3′ poly(A) tail. It consists of two open reading frames, which respectively encode four nonstructural proteins (nsP1-4) and six structural proteins (capsid, E3, E2, 6K, transframe, and E1) (12). After release into the cytosol from internalized virions, G RNA is directly translated to produce the nonstructural polyprotein precursors P1234 and P123, which are processed by the protease activity of nsP2 into individual nsPs (13). While nsP4 is the RNA-dependent RNA polymerase, all four nsPs are required for replication of viral G RNA (14, 15). These nsPs interact with viral RNA as well as multiple host components and form plasma membrane-bound viral replication complexes (vRCs) known as spherules (16–18). The vRCs are responsible for the synthesis of G RNA and subgenomic viral RNA, which serves as a template for the translation of structural proteins. These proteins ultimately assemble with G RNA to form progeny virions (19).

Alphavirus nsP3 has been suggested to play an important role in assembly of the vRC. nsP3 contains an N-terminal macro domain, an alphavirus unique domain, and a C-terminal hypervariable domain (HVD). The HVD is intrinsically disordered and contains linear motifs which serve as hubs for recruitment of cellular factors that are required for vRC formation and function (20). Several cellular proteins that interact with alphavirus nsP3 have been identified (21, 22). The best studied ones are the fragile X-related (FXR) and Ras-GTPase-activating protein SH3 domain-binding protein (G3BP) family members (23–25). It has been shown that the HVDs of NWs interact with all members of the FXR family, while HVDs of OWs recruit G3BP1 and G3BP2 of the G3BP family (23). Other HVD-binding proteins with redundant functions in alphavirus replication include FHL and NAP1 family members, and the SH3 domain-containing proteins CD2AP, SH3KBP1, and BIN1, which are proviral but not critical for viral replication (26–28). However, despite intensive investigations, the molecular mechanisms of alphavirus replication, especially its regulation by the host, are still enigmatic.

In this study, we identified Y-box binding protein 1 (YBX1), a member of the DNA/RNA-binding protein family with a conserved cold shock domain (CSD) (29), as a critical cellular factor for alphaviruses. Unlike the previously characterized host factors which are important mostly for a unique alphavirus (23), YBX1 plays a key role in the replication of both NWs and OWs. Our findings suggest that YBX1 may serve as a promising target for drug development against multiple alphaviruses.

## RESULTS

### Deficiency of YBX1 impairs replication of multiple alphaviruses

**Fig 1:**
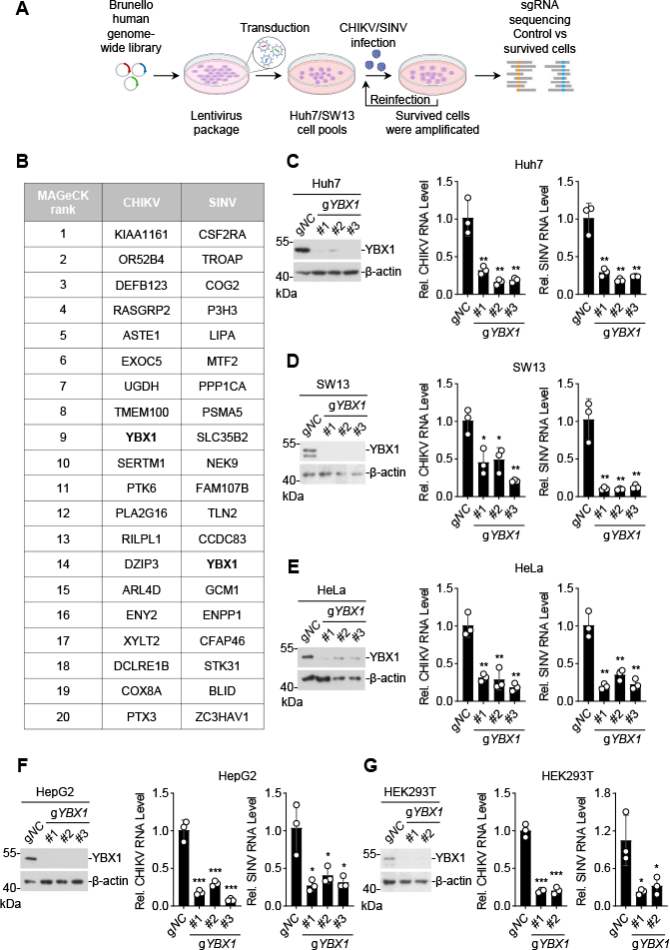
Identification of YBX1 as an important host factor for CHIKV/SINV by genome-wide CRISPR/Cas9 screens. (**A**) A schematic diagram of the genome-wide CRISPR/Cas9 screen process. (**B**) Statistical chart of the screen results of CHIKV and SINV infection. The top 20 candidates ranked by the MAGeCK score are shown in the table. (**C–G**) Effects of YBX1 deficiency on CHIKV or SINV infection in various cells. Huh7 (**C**), SW13 (**D**), HeLa (**E**), HepG2 (**F**), and HEK293T (**G**) cells were edited with a control gRNA or the indicated numbers of gRNAs targeting the *YBX1* gene. Cells were infected with CHIKV (multiplicity of infection [MOI] = 1) or SINV (MOI = 1) for 12 h before reverse transcription quantitative PCR was performed. Data are normalized to the CHIKV RNA level or SINV RNA level in the control gRNA-edited cells. Data are represented as mean ± SD. *P < 0.05, **P < 0.01, ***P < 0.001.

To identify cellular factors critical for infection and replication of alphaviruses, we performed genome-wide CRISPR/Cas9 screens using human hepatocellular carcinoma Huh7 cells and human adrenal carcinoma SW13 cells for CHIKV (181/25) and SINV (AR339) infections, respectively (Fig. 1A). Huh7 and SW13 cells are highly susceptible to CHIKV and SINV, respectively, and almost all cells died within 3 days after infection. After two rounds of infection, the survived cells were analyzed for edited genes. The robust rank aggregation algorithm of MAGeCK software was used to sort the differentially enriched edited genes between the control and selected samples (30, 31). As shown in Fig. 1B, YBX1 was identified among the top 20 candidate genes from screens with either CHIKV or SINV. Studies have demonstrated that YBX1 is involved in various biological process such as transcription, mRNA splicing, processing of noncoding RNA, regulation of mRNA stability, and DNA damage repair (32). A previous study has also identified YBX1 as a candidate protein in screens for cellular factors required for replication of CHIKV (26), but neither validated YBX1 as a cellular factor for CHIKV nor investigated the mechanisms involved. Therefore, we first validated YBX1 function on CHIKV and SINV replication in various cells by CRISPR/Cas9. The results indicated that viral RNA levels of CHIKV and SINV were significantly decreased in *YBX1*-edited Huh7, SW13, human cervical carcinoma HeLa, human liver cancer HepG2, and human embryonic kidney 293T (HEK293T) cells (Fig. 1C through G). A highly effective YBX1 inhibitor, SU056, also inhibited CHIKV replication in a dose-dependent manner in baby hamster kidney BHK-21 cells (Fig. S1).

**Fig 2:**
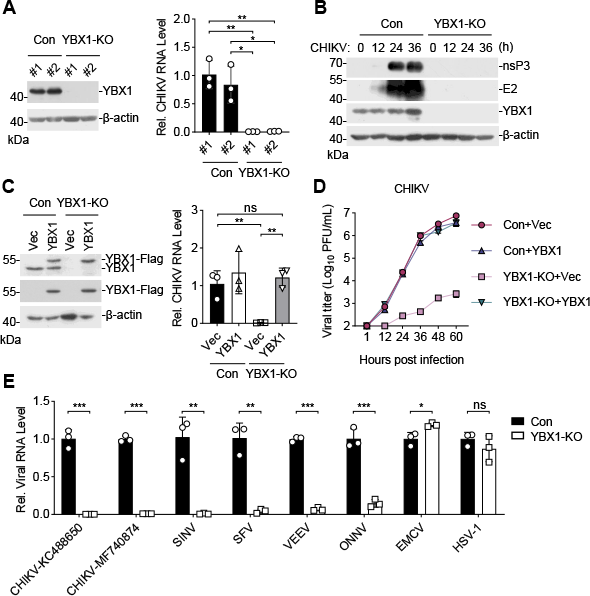
YBX1 is required for the efficient infection of multiple alphaviruses. (**A and B**) CHIKV infectivity in YBX1-knockout HeLa cells. Single clones of YBX1-knockout HeLa cells were isolated and confirmed by immunoblots (A, left panel). The control (Con) or YBX1-deficient (YBX1-KO) clones were infected with CHIKV (MOI = 1) for the indicated times. RNA level of CHIKV (A, right panel; 12 hpi) and CHIKV protein expression (B; 12, 24, and 36 hpi) were measured by RT-qPCR and immunoblots, respectively. (**C and D**) Reconstitution of YBX1-deficient cells with YBX1 restores CHIKV infection. The Con or YBX1-KO HeLa cells reconstituted with empty vector or YBX1-Flag were infected with CHIKV (MOI = 1) for the indicated times. The expression of YBX1, RNA level of CHIKV, and production of CHIKV progeny viruses were measured by immunoblots (C, left panel), RT-qPCR (C, right panel), and plaque assay (**D**), respectively. (**E**) Effects of YBX1 deficiency on CHIKV-KC488650, CHIKV-MF740874, SINV, SFV, VEEV, ONNV, EMCV, and HSV-1 infection. The Con or YBX1-KO HeLa cells were inoculated with the indicated viruses at an MOI of 1 for 12 h (SINV, SFV, VEEV, ONNV, EMCV, and HSV-1) or 24 h (CHIKV-KC488650 and CHIKV-MF740874) before RT-qPCR was performed. Data are normalized to the viral RNA level in the control cells. Data are represented as mean ± SD. *P < 0.05, **P < 0.01, ***P < 0.001. ns, not significant.

In the above experiments, *YBX1*-edited cell pools were used for CHIKV and SINV infection, and the failed editing of a fraction of cells in the pools may be responsible for the residual replication of the viruses. To determine whether YBX1 is required for replication of alphaviruses, we isolated single clones of *YBX1*-edited HeLa cells in which YBX1 expression was completely deficient (Fig. 2A). After CHIKV infection, the viral RNA (Fig. 2A) as well as nsP3 and E2 proteins (Fig. 2B) were barely detectable in cells deficient of YBX1. Trans-complementation of YBX1-deficient HeLa cells with a human cDNA encoding YBX1 restored CHIKV RNA replication (Fig. 2C) and the release of the progeny viruses (Fig. 2D). We next determined whether YBX1 is required for replication of other alphaviruses. Reverse transcription quantitative PCR (RT-qPCR) results indicated that replication of all the examined alphaviruses was impaired in YBX1-deficient cells including CHIKV-KC488650 (an Asian strain from a clinically CHIKV-positive patient in China), CHIKV-MF740874 (a strain of the East/Central/South African genotypes), SINV, SFV, VEEV, and ONNV, whereas YBX1 deficiency did not impair replication of encephalomyocarditis virus (EMCV) or herpes simplex virus 1 (HSV-1) (Fig. 2E). These results suggest that YBX1 is specifically required for replication of alphaviruses.

### YBX1 is required for the formation of vRCs after CHIKV infection

**Fig 3:**
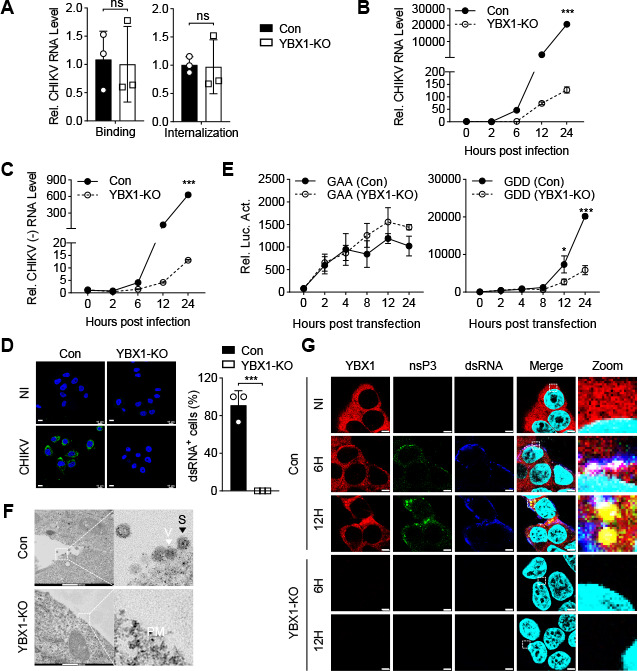
YBX1 is essential for viral RNA replication. (**A**) Effects of YBX1 on CHIKV binding and internalization. The control (Con) or YBX1-deficient (YBX1-KO) HeLa cells were incubated with CHIKV (MOI = 10) on ice (binding) or at 37°C (internalization) as described in Materials and Methods. The CHIKV RNA levels were measured by RT-qPCR. Data are normalized to the CHIKV RNA level in the control cells. (**B and C**) Effects of YBX1 deficiency on CHIKV total RNA or (−) RNA levels produced during virus replication. The Con or YBX1-KO HeLa cells were infected with CHIKV (MOI = 1) for the indicated times. The cells were collected, and CHIKV total RNA (**B**) or (−) RNA (**C**) level was measured by RT-qPCR. (**D**) Effects of YBX1 deficiency on CHIKV dsRNA intermediates produced during virus replication. The Con or YBX1-KO HeLa cells were infected with CHIKV (MOI = 50) for 16 h and then stained with anti-dsRNA J2 monoclonal antibody (left). The statistical results of positive cells are shown on the right. Scale bars, 10 µm. (**E**) Effects of YBX1 on CHIKV translation and replication. The Con or YBX1-KO HEK293T cells were transfected with *in vitro* transcribed wild-type (GDD, CHIKV-nsP3-Fluc) or replication-deficient (GAA, CHIKV-nsP3-Fluc-nsP4^GAA^) RNA. The firefly luciferase activity was assessed at the indicated times. (**F**) Effects of YBX1 on CHIKV-induced formation of plasma membrane (PM)-associated spherules (S). The Con or YBX1-KO HEK293T cells were incubated with CHIKV-181/25 (MOI of 50) for 18 h before transmission electron microscopy analyses. Replication S, together with viral particles (V) at the PM. Scale bars, 500 nm. (**G**) YBX1 efficiently accumulates in large cytoplasmic CHIKV nsP3-containing complexes. The Con or YBX1-KO HEK293T cells were incubated with CHIKV-181/25-nsP3-GFP (MOI of 50) for the indicated times, then fixed with 4% paraformaldehyde and stained with indicated antibodies before confocal microscopy. The areas in the dashed boxes on the left are enlarged on the right. Images are representative of three experiments. Scale bars, 5 µm. Data are represented as mean ± SD. *P < 0.05, ***P < 0.001. ns, not significant.

We next investigated the mechanism on how YBX1 is required for replication of alphaviruses. We challenged YBX1-deficient and control HeLa cells with CHIKV and performed binding and internalization assays. The results indicated that deficiency of YBX1 did not affect CHIKV binding or internalization (Fig. 3A), suggesting that YBX1 is dispensable for CHIKV entry. We then evaluated the effects of YBX1 on viral RNA replication by measuring viral RNA levels at different time upon CHIKV infection. The results indicated that CHIKV RNA levels dramatically increased from 6 to 24 h post-infection in control HeLa cells, whereas they were only minimally increased in YBX1-deficient cells from 6 to 24 h post-infection (Fig. 3B). Given that the synthesis of negative-strand RNA [(−) RNA] is the first step of CHIKV RNA replication and the production of dsRNA intermediates is a marker of viral replication complex assembly, we investigated the effects of YBX1 on these two indicators of CHIKV RNA replication. Strand-specific RT-qPCR and immunofluorescence experiments showed that both CHIKV (−) RNA and dsRNA intermediates were severely reduced in YBX1-deficient HeLa cells compared with control cells (Fig. 3C and D). These results suggest that YBX1 is required for synthesis of viral (−) RNA upon CHIKV infection.

To further evaluate the contribution of YBX1 in viral RNA translation versus replication, we generated wild-type (CHIKV-nsP3-Fluc) and replication-deficient (CHIKV-nsP3-Fluc-nsP4^GAA^, in which the GDD motif of the polymerase nsP4 was mutated to GAA, rendered the vRNA permissive for translation but deficient for replication) CHIKV RNA, in which a firefly luciferase (Fluc) was in-frame fused to the C-terminus of nsP3. Transfection of translation-permissive but replication-deficient CHIKV RNA (CHIKV-nsP3-Fluc-nsP4^GAA^) resulted in similar Fluc activities in YBX1-deficient and control HEK293T cells, suggesting that YBX1 was dispensable for viral RNA translation. In similar experiments, transfection of wild-type CHIKV RNA (CHIKV-nsP3-Fluc) in YBX1-deficient HEK293T cells resulted in markedly impaired Fluc signals compared with control cells, indicating that YBX1 was required for viral RNA replication (Fig. 3E). Consistently, transmission electron microscopy showed that vRCs such as the plasma membrane-associated spherules were observed in control but not YBX1-deficent HEK293T cells (Fig. 3F). Furthermore, immunofluorescent microscopy indicated that YBX1 was diffusely distributed in the cytoplasm of noninfected cells. However, in cells infected with CHIKV-nsP3-GFP (a reporter virus expressing GFP that is in-frame fused to the C-terminus of nsP3) for 6 h, YBX1 formed dot-like aggregates overlapped with nsP3-GFP and dsRNA (Fig. 3G). Interestingly, these dot-like aggregates were mostly localized at the plasma membrane at 6 h post-infection. At 12 h post-infection, YBX1 formed bigger nsP3-GFP overlapping aggregates that were mostly localized in the cytoplasm. In YBX1-deficient cells, nsP3- and dsRNA-positive aggregates were not observed either at 6 nor 12 h post-infection. These results suggest that YBX1 is required for the formation of vRCs and replication of CHIKV.

### Interaction of nsP3 with YBX1 is required for CHIKV replication

**Fig 4:**
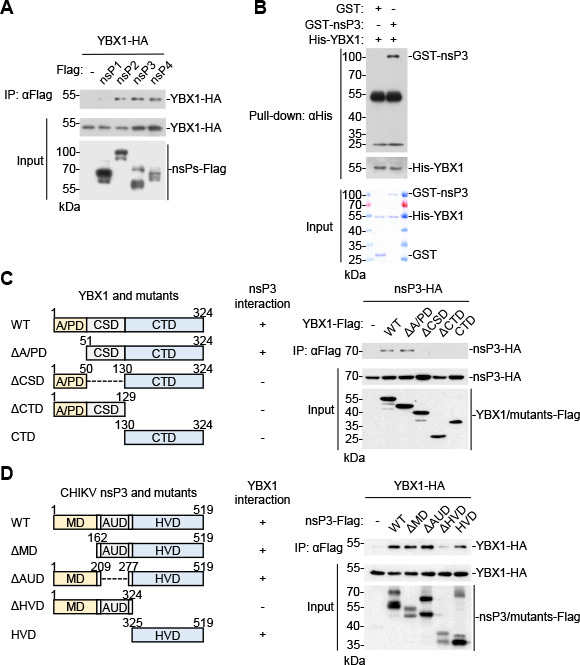
YBX1 directly interacts with CHIKV nsP3 through CSD and C-terminal domain (CTD) of YBX1 and HVD of CHIKV nsP3. (**A**) YBX1 interacts with CHIKV nsPs in the mammalian overexpression system. HEK293T cells were transfected with indicated plasmids for 24 h. Co-immunoprecipitation and immunoblot analysis were performed with the indicated antibodies. (**B**) Direct interaction between YBX1 and CHIKV nsP3. Purified His-YBX1 and GST-nsP3 were incubated as indicated. Pull-down was performed with Ni magnetic beads, and the proteins present in the pellet were denatured and subjected to SDS-PAGE and immunoblotting with the indicated antibodies. (**C and D**) Domain mapping of YBX1-nsP3 interaction. HEK293T cells were transfected with the indicated plasmids for 24 h before co-immunoprecipitation and immunoblot analysis with the indicated antibodies. The schematic representations of YBX1 and nsP3 truncations are shown on the left.

We next investigated how YBX1 is involved in vRC formation and viral replication upon CHIKV infection. We first examined whether YBX1 interacts with the components of vRCs. Co-immunoprecipitation experiments showed that YBX1 interacted with all four CHIKV nsPs, with the weakest interaction with nsP1 (Fig. 4A). As a previous study shows that YBX1 was identified in SINV nsP3-specific protein complexes by mass spectrometry (33), we then determined whether YBX1 was recruited into vRCs by directly interacting with CHIKV nsP3. We prepared recombinant YBX1 and CHIKV nsP3 in *Escherichia coli*, and *in vitro* pull-down assays showed that YBX1 directly interacted with CHIKV nsP3 (Fig. 4B).

YBX1 contains an N-terminal Ala/Pro-rich domain (A/PD), a CSD in the middle and a C-terminal domain (CTD) consisting of alternating base/acid amino acid (aa) repeats (29). The function of YBX1 N-terminal A/PD is unknown. The CSD of YBX1 is conserved in evolution from prokaryotes to vertebrates and recognizes DNA/RNA (32). Domain mapping and co-immunoprecipitation experiments indicated that the HVD of CHIKV and SINV nsP3, as well as both CSD and CTD domains of YBX1, was required for their interaction (Fig. 4C and D; Fig. S2). These data suggest that YBX1 interacts with CHIKV nsP3.

**Fig 5:**
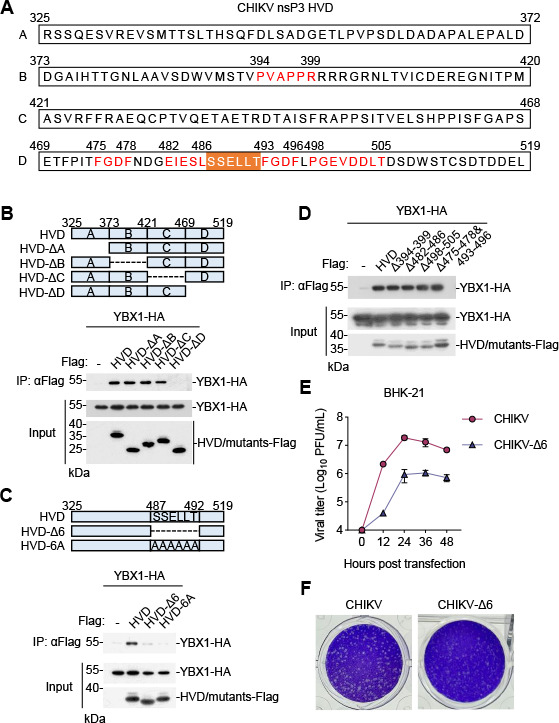
Mutations in the YBX1-binding site of nsP3 HVD attenuate CHIKV replication. (**A**) A schematic diagram of Flag-HVD cassettes applied for mapping of YBX1-binding sites. (**B**) YBX1-binding sites are located in the D fragment of the CHIKV HVD. HEK293T cells were transfected with the indicated plasmids for 24 h. Co-immunoprecipitation and immunoblot analysis were performed with the indicated antibodies. The schematic representations of YBX1 mutations are shown at the top. (**C**) The peptide located between aa 487 and 492 of CHIKV HVD is a potential YBX1-binding site. The experiments were similarly performed as in panel** B **except that different YBX1 mutants were used. (**D**) The four conserved motifs of CHIKV nsP3 HVD are not responsible for interaction with YBX1. The experiments were similarly performed as in panel **B **except that different YBX1 mutants were used. (**E and F**) Deletion of the YBX1-binding site in the CHIKV genome attenuates CHIKV replication. BHK-21 cells were transfected with CHIKV and CHIKV-Δ6 RNA, respectively. The supernatants were harvested at the indicated times. Production of progeny viruses was measured by plaque assay (**E**). The representative plaques were photographed and recorded (**F**).

To map the residues of nsP3 that are important for its interaction with YBX1, we constructed four deletion mutants of CHIKV nsP3 HVD; in each, a fragment of 48 or 51 aa residues in HVD of nsP3 was deleted (Fig. 5A and B). Co-immunoprecipitation experiments indicated that deletion of the C-terminal 51 aa of nsP3 HVD (HVD-ΔD), but not the other examined deletions, abolished the interaction of nsP3 with YBX1 (Fig. 5B). Further deletion analysis indicated that deletion of six residues (aa 487–492) in the HVD of nsP3 (HVD-Δ6) or mutation of the six residues to alanine (HVD-6A) impaired its interaction with YBX1 (Fig. 5C). Interestingly, deletion of two conserved motifs (aa 482–486 and aa 498–505), as well as motifs “PVAPPR” (aa 394–399) and “FGDF” (aa 475–478 and aa 493–496) which have been shown to be responsible for nsP3 interaction with Bin1 and G3BP, respectively (21), did not affect its interaction with YBX1 (Fig. 5D). These results suggest that nsP3 interacts with YBX1 via a unique motif (SSELLT), which has not been reported to interact with other host factors before. Since this YBX1-binding motif exists only in CHIKV but not SINV, SFV, ONNV, or VEEV, it is likely that the binding sites of nsP3 to YBX1 vary among different alphaviruses.

To further validate the role of nsP3-YBX1 interaction in CHIKV replication, we generated a CHIKV infectious clone (CHIKV-Δ6) in which the six residues (aa 487–492) of nsP3 were deleted and prepared *in vitro*-synthesized CHIKV-Δ6 RNA. We transfected wild-type and CHIKV-Δ6 RNA into BHK-21 cells and then measured viruses released from the transfected cells. The results indicated that cells transfected with CHIKV-Δ6 RNA produced ~10-fold less viruses (Fig. 5E). Moreover, the plaques formed by CHIKV-Δ6 infection were markedly smaller than those formed by wild-type CHIKV infection (Fig. 5F). These data further support that binding of nsP3 to YBX1 is important for CHIKV replication. It is surprising that YBX1 deficiency also impaired the replication of CHIKV-Δ6 virus (Fig. S3). However, since the interaction between YBX1 and nsP3-HVD-Δ6 was weakened but not completely lost (Fig. 5C), one explanation is that the remaining interaction between YBX1 and CHIKV-nsP3-Δ6 may be responsible for the differential replication of CHIKV-Δ6 virus in control versus YBX1-KO cells.

It has been reported that YBX1 redistributed with SINV capsid early in infection (34). To further test whether it contributes to the function of YBX1 on CHIKV replication, we generated a CHIKV-derived replicon in which the structural genes in the viral genome were replaced with the *Renilla* luciferase gene. The results showed that YBX1 deficiency also impaired replication of the replicon (Fig. S4), indicating the function of YBX1 on CHIKV replication is capsid independent.

### Binding of viral RNA to YBX1 is required for efficient replication of CHIKV

**Fig 6:**
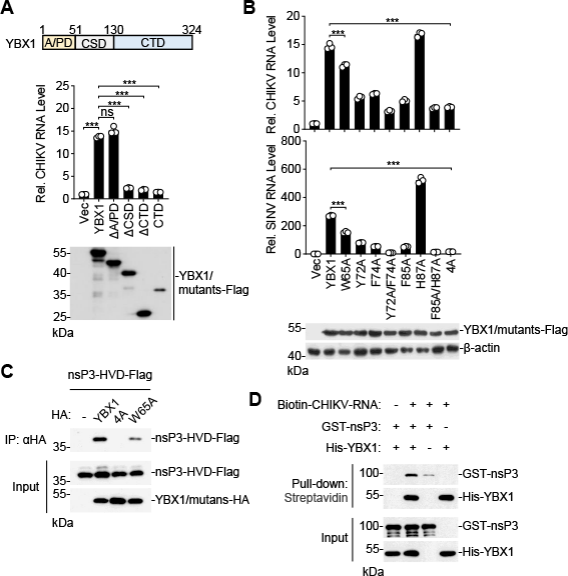
RNA-binding activity of YBX1 is required for efficient virus replication. (**A**) The CSD and CTD domains of YBX1 are important for CHIKV replication. YBX1-deﬁcient (YBX1-KO) HeLa cells reconstituted with empty vector, YBX1-Flag, or the indicated YBX1 truncations were inoculated with CHIKV (MOI = 1) for 12 h before RT-qPCR analysis. The structural schematic representations of YBX1 are shown at the top. (**B**) RNA-binding ability of YBX1 is required for infection of CHIKV and SINV. YBX1-KO HeLa cells reconstituted with empty vector, YBX1-Flag, or the indicated YBX1 mutations were inoculated with CHIKV (MOI = 1) or SINV (MOI = 1) for 12 h before RT-qPCR analysis. 4A, Y72A/F74A/F85A/ H87A. For bar graphs, data are normalized to that of the vector-reconstituted cells. (**C**) RNA-binding ability of YBX1 is required for interaction with CHIKV nsP3. HEK293T cells were transfected with the indicated plasmids for 24 h. Co-immunoprecipitation and immunoblot analysis were performed with the indicated antibodies. (**D**) YBX1 promotes the binding of CHIKV nsP3 to CHIKV RNA. Purified His-YBX1, GST-nsP3, and biotin-labeled CHIKV RNA were incubated as indicated. Pull-down was performed with streptavidin magnetic beads, and the proteins present in the pellet were denatured and subjected to SDS-PAGE and immunoblotting with the indicated antibodies. Data are represented as mean ± SD. ***P < 0.001. ns, not significant.

To determine whether the ability of YBX1 to bind nucleic acids is necessary for CHIKV replication, we reconstituted YBX1-deficient HeLa cells with wild-type YBX1 and its various mutants. Trans-complementation of YBX1-deficient cells with wild-type YBX1 or YBX1-∆A/PD, but not YBX1-∆CSD, YBX1-∆CTD, or YBX1-CTD, rescued CHIKV replication (Fig. 6A), suggesting that both CSD and CTD but not the N-terminal A/PD of YBX1 were required for replication of CHIKV. YBX1 contains two RNA-binding motifs in the CSD, RNP1 (GYGFI, aa 71–75) and RNP2 (VFVHQ, aa 84–88), which are characteristic of many RNA-binding proteins (29, 35). We constructed various point mutations of the key amino acid residues involved in RNA binding, including W65A, Y72A, F74A, Y72A/F74A, F85A, H87A, F85A/H87A, and Y72A/F74A /F85A/H87A (4A). Trans-complementation of YBX1-deficient cells with all the YBX1 mutants, except YBX1^H87A^, showed a significant decrease in CHIKV and SINV replication in comparison to cells trans-complemented with wild-type YBX1 (Fig. 6B), suggesting the RNA-binding activity of YBX1 is important for CHIKV replication. We then examined whether the RNA-binding ability is required for its interaction with CHIKV nsP3. Co-immunoprecipitation experiments indicated that YBX1^4A^ failed to bind CHIKV nsP3-HVD, while the binding of YBX1^W65A^ with CHIKV nsP3-HVD was markedly reduced (Fig. 6C). Notably, the ability of these mutants to impair the interaction between YBX1 and nsP3-HVD correlated with their ability to inhibit replication of CHIKV or SINV. These results suggest that the RNA-binding activity of YBX1 is indispensable for its ability to bind CHIKV nsP3 and conditioning for viral replication.

We next examined the direct interaction among YBX1, nsP3, and CHIKV RNA by *in vitro* pull-down assays. As shown in Fig. 6D, both YBX1 and CHIKV nsP3 bound to CHIKV RNA and YBX1 enhanced the binding of CHIKV nsP3 to CHIKV RNA.

### YBX1 facilitates CHIKV replication independently of RNA 5-methylcytosine modification

**Fig 7:**
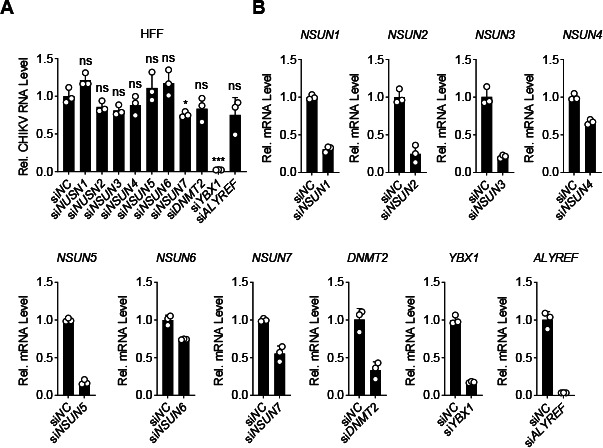
The replication of CHIKV is independent of m5C machinery. (**A and B**) HFF cells were transfected with the indicated siRNAs for 48 h and then infected with CHIKV-181/25 (MOI = 1) for 12 h before RT-qPCR analysis. Data are normalized to that of control siRNA-transfected cells. Data are represented as mean ± SD. *P < 0.05, ***P < 0.001. ns, not significant.

Since YBX1 is a 5-methylcytosine (m^5^C) RNA reader (36), we next determined whether RNA m^5^C modification is involved in CHIKV replication. We knocked down the expression of known writers, readers of RNA m^5^C methylation, and other members of NOL1/NOP2/SUN RNA methyltransferase family in human foreskin fibroblast (HFF) cells using siRNAs and examined their effects on CHIKV replication. As shown in new Fig. 7A and B, while knockdown of YBX1 significantly impaired CHIKV replication, knockdown of other m^5^C writers or readers showed no obvious effects. In addition, it is noteworthy that W65 of YBX1 has been reported to be a key residue for m^5^C binding, while it also contributes to binding to unmodified RNA (37). In YBX1-deficient cells, the mutant YBX1^W65A^ restored CHIKV infection to ~80% of that in cells reconstituted with wild-type YBX1 (Fig. 6B), suggesting that the function of YBX1 as an m^5^C reader is not essential for its effect on CHIKV replication.

## DISCUSSION

Identification of cellular factors required for replication of alphaviruses is important for understanding the life cycles of these notorious viruses and providing potential targets for intervention of the related infectious diseases. Previous studies demonstrate that the intrinsically disordered HVDs of alphavirus nsP3 play indispensable roles in the formation of vRCs, and the HVDs of various alphaviruses interact with distinct cellular factors which are differentially required for assembly of vRCs upon infection of different alphaviruses (20–24). In this study, we have demonstrated that YBX1 is a critical cellular factor for assembly of vRCs of CHIKV and replication of multiple alphaviruses. We have also shown that YBX1 is not required for replication of other examined types of viruses, including EMCV and HSV-1, suggesting that YBX1 is specifically required for replication of alphaviruses. Our experiments demonstrate that YBX1 is not required for CHIKV binding to and internalization into the host cells but for assembly of plasma membrane-associated vRCs after CHIKV infection. Co-immunoprecipitation and *in vitro* pull-down assays indicate that YBX1 binds to both CHIKV RNA and nsP3 via its RNA-binding motifs in CSD and CTD domains. Further experiments demonstrate that YBX1 promotes the binding of nsP3 to CHIKV RNA. Deficiency of YBX1, mutation of the six aa nsP3 motif responsible for its binding to YBX1, and mutation of the YBX1 RNA-binding motifs all impair CHIKV replication. Taken together, these experiments suggest that YBX1 may act as a scaffold for assembly of vRCs upon infection of alphaviruses. How YBX1 is recruited to vRCs upon CHIKV infection needs additional investigation.

Previously, it has been demonstrated that nsP3 of various alphaviruses interacts with G3BP and FXR family proteins, which are differentially required for replication of distinct alphaviruses (23). Despite low sequence homology at the amino acid level, YBX1 shares certain properties with the G3BP and FXR family proteins, such as the presence of RNA-binding motifs and involvement in the formation of ribonucleoprotein complexes, including stress granules (SGs) (38, 39). It has been shown that CHIKV and SINV nsP3 HVDs interact with both G3BP1 and G3BP2 but not with FXRs, while the VEEV nsP3 HVD binds all three FXRs but not G3BPs (23). Our mutational analysis indicates that the six aa motif “SSELLT” of CHIKV nsP3 HVD is required for its binding with YBX1. However, YBX1 deficiency also impaired replication of CHIKV-Δ6 virus. One explanation is that the remaining interaction between YBX1 and CHIKV-nsP3-Δ6 may play a role in the replication of CHIKV-Δ6 virus in control cells. Alternatively, the interaction between YBX1 and nsP2 or nsP4 may contribute to the replication of CHIKV-Δ6 virus. Although YBX1 binds to the HVDs of both CHIKV and SINV nsP3, sequence analysis suggests that the SSELLT binding motif of CHIKV is not conserved among the alphaviruses. It is possible that different alphaviruses utilize different motifs for YBX1 binding. Further investigation is needed to explore the binding motifs in nsP3 of other alphaviruses.

YBX1 has been demonstrated as a m^5^C RNA reader. However, such function of YBX1 is dispensable for its effect on CHIKV replication. First, replication of CHIKV was barely affected when proteins of the m^5^C machinery, except YBX1, were knocked down. Second, in YBX1-deficient cells, YBX1^W65A^ restored CHIKV infection to ~80% of that in cells reconstituted with wild-type YBX1. The slight decrease may be due to the weakened ability of YBX1^W65A^ to bind to unmodified RNA. The RNA m^5^C modification of CHIKV and other alphaviruses needs further confirmation by RNA epitranscriptome sequencing.

In addition to alphaviruses, YBX1 is also involved in regulation of life cycles of other viruses. For example, it has been demonstrated that YBX1 interacts with dengue virus RNA and is required for efficient assembly of intracellular infectious virions and their secretion (40). YBX1 has also been reported to interact with the NS3/4A and vRNA of hepatitis C virus (HCV) to modulate the equilibrium between HCV RNA replication and the production of infectious particles (41, 42). In addition, YBX1 has been shown to stabilize human immunodeficiency virus type 1 (HIV-1) RNA in producer cells and increase virus production (43). Moreover, it promotes mouse mammary tumor virus assembly by facilitating the formation of intracytoplasmic Gag-RNA complexes (44). The RNA-binding specificity of YBX1 is not well defined, although it has been reported that YBX1 prefers binding to a CAUC consensus (35). Whether the function of YBX1 in alphavirus replication relies on the ability of YBX1 to bind specific alphavirus RNA sequences awaits further investigation. Nevertheless, our findings reveal the first cellular factor that is required for both the NW and OW alphaviruses, which may serve as a target for development of drugs for pan-alphaviruses.

## MATERIALS AND METHODS

### Cells

SW13, HeLa, HepG2, HEK293T, Vero E6 (American Type Culture Collection), Huh7 (provided by the National Virus Resource Center, Wuhan, China), and BHK-21 (provided by Bo Zhang, Wuhan Institute of Virology, Chinese Academy of Sciences [CAS]) cells were cultured at 37°C in Dulbecco’s Modified Eagle Medium (Gibco) supplemented with 10% (vol/vol) fetal bovine serum and 1% (vol/vol) penicillin-streptomycin (Hyclone). All cell lines were tested and conformed to be free from mycoplasma contamination using the MycoBlue Mycoplasma Detector kit (#D101, Vazyme).

### Viruses

CHIKV (Asian strain, JC2012, KC488650.1) and CHIKV (ESCA strain, Pakistan-03, MF740874.1) were provided by the National Virus Resource Center. SINV (strain AR339, OK539682.1) and VEEV (strain TC-83, L01443.1) were provided by Dr. Bo Zhang (Wuhan Institute of Virology, CAS). SFV (strain SFV4, KP699763.1) was provided by Dr. Xi Zhou (Wuhan Institute of Virology, CAS). HSV-1 (KOS strain) was provided by the China Center for Type Culture Collection. EMCV (BJC3 strain) was provided by Dr. Han-Chun Yang (China Agricultural University). CHIKV-181/25 (MW473668.1), CHIKV-181/25-GFP, CHIKV-181/25-nsP3-GFP, and ONNV (Gulu strain, M20303.1) were rescued from pACYC177-vector-based cDNA clones. The alphaviruses EMCV and HSV-1 were propagated in Vero E6 cells and titrated by standard plaque assays.

### RNA transcription, transfection, and virus rescue

Viral RNAs were prepared from the BamHI-linearized cDNA plasmids through *in vitro* transcription using mMESSENGER mMACHINE T7 Kit (#AM1344, Invitrogen) and transfected into BHK-21 cells with DMRIE-C (#10459014, Invitrogen). At different time points post-transfection, supernatants were collected. The culture medium containing viruses were aliquoted and stored at −80°C.

### Antibodies and reagents

Rabbit anti-YBX1 polyclonal antibody (#20339–1-AP, Proteintech), rabbit anti-β-actin monoclonal antibody (#AC026, ABclonal), anti-HA-tag mAb-HRP-DirecT (#M180-7, MBL), anti-Flag-tag mAb-HRP-DirecT (#M185-7, MBL), GST-tag (26H1) Mouse mAb (#2624, Cell Signaling Technology), His-tag (D3I1O) XP Rabbit mAb (#12698, Cell Signaling Technology), mouse anti-dsRNA J2 monoclonal antibody (#10010200, Nordic MUbio), Alexa Fluor 488-conjugated donkey anti-mouse IgG (#R37114, Thermo Fisher), Alexa Fluor 555-conjugated goat anti-rabbit IgG (#A21429, Thermo Fisher), and Alexa Fluor 647-conjugated goat anti-mouse IgG (#A21235, Thermo Fisher) were purchased from the indicated companies. Mouse anti-CHIKV nsP3 and mouse anti-CHIKV E2 antibodies were generated by immunization of BALB/c mice with puriﬁed CHIKV nsP3 and E2 proteins, respectively. SU056 (#HY-150231, MedChemExpress) was purchased from the indicated manufacturer.

### Constructs

The coding sequence of CHIKV-181/25 was synthesized (Sangon Biotech) and cloned into the vector pACYC177 by homologous recombination. Based on this plasmid, pACYC177-CHKV-181/25-GFP was constructed by inserting a second SG promoter, a linker sequence and the GFP gene after the 3′-terminal of E1 gene. pACYC177-CHIKV-181/25-nsP3-GFP and pACYC177-CHIKV-181/25-nsP3-Fluc were constructed by inserting the *GFP* gene and *Fluc* gene after codon 490 of the *nsP3* gene, respectively. An inactivating GDD to GAA mutation in the catalytic site of nsP4 was introduced by PCR-mediated mutagenesis to obtain the pACYC177-CHIKV-181/25-nsP3-Fluc-nsP4^GAA^. The CHIKV-derived replicon was provided by Dr. Bo Zhang (Wuhan Institute of Virology, CAS). The C-terminal Flag-tagged CHIKV nsP1, nsP2, nsP3, and nsP4 plasmids were constructed into the pLOV-CMV-Puro vector. The nsP3 truncations were constructed by PCR-mediated mutagenesis of pLOV-CMV-Puro-nsP3. The C-terminal HA-tagged CHIKV nsP3 and YBX1 plasmids were constructed into the pRK vector. The C-terminal Flag-tagged YBX1 plasmid was constructed into the pLOV-CMV-Blast vector. The Flag-tagged truncations and mutations of YBX1 were constructed at the foundation of pLOV-CMV-Blast-YBX1. For trans-complementation experiments, the YBX1 sgRNA targeting sequences were mutated synonymously in the plasmids to avoid re-cutting by Cas9. The His-tagged YBX1 was constructed into the pET30c vector. The GST-tagged CHIKV nsP3 was constructed into the pGEX vector. All plasmids were constructed by standard molecular biology techniques.

### CRISPR genetic screen

The human CRISPR Brunello lentiviral pooled libraries (#73179, Addgene) encompassing 76,441 different gRNAs targeting 19,114 genes were purchased from Addgene. Lentiviral production was prepared in HEK293T cells by co-transfecting gRNA plasmids with psPAX2 and pMD2.G. Supernatants were collected 48 h after transfection. Huh7 and SW13 cells were transduced with CRISPR-sgRNA lentiviral library at a multiplicity of infection (MOI) of 0.3. Cells were selected with puromycin for 7 days and expanded. Then, 100 million cells were pooled and inoculated with CHIKV-181/25 (MOI of 1) and SINV-AR336 (MOI of 1), respectively. Approximately 2–3 days after infection, cytopathic effects were detectable, and surviving cells were collected 2 weeks later. After two rounds of infection, genomic DNA was extracted from selected cells or uninfected pooled cells using a TIANamp Genomic DNA Kit (TIANGEN), and inserted gRNA sequences were amplified and sequenced using next-generation sequencing on an Illumina Seq platform (Azenta). gRNA sequences were analyzed using the MAGeCK software.

### CRISPR/Cas9 knockout

Gene editing was performed with the CRISPR/Cas9 system. Brieﬂy, double-stranded oligonucleotides corresponding to the target sequences were cloned into the lenti-CRISPR-v2 vector, which was co-transfected with the packaging plasmids psPAX2 and pMD2.G into HEK293T cells. Two days after transfection, lentiviruses were harvested and used to infect target cells. The infected cells were selected with puromycin (1 µg/mL) for at least 7 days. Three different gRNAs targeting *YBX1* were used: g*YBX1*-1 (5′-GGCGGGGACAAGAAGGTCAT-3′), g*YBX1*-2 (5′-GCAAATGTTACAGGTCCTGG-3′), and g*YBX1*-3 (5′-GTCTTGCAGGAATGACACCA-3′). The YBX1-deﬁcient clones were obtained by limited dilution. *YBX1* gene mutation and its deﬁciency were conﬁrmed by Sanger sequencing and immunoblots, respectively.

### RNA interference

The siRNAs (RiboBio, Guangzhou, China) were transfected into HFF cells using Lipofectamine RNAiMAX transfection reagent (#13778, Invitrogen) following the manufacturer’s instructions. Forty-eight hours after transfection, the medium was replaced with fresh medium and cells were infected with CHIKV. The sequences of siRNA oligonucleotides are included in the supplemental information (Table S1).

### RT-qPCR

Total RNA from the cells was isolated with RNAiso Plus (#9109, TaKaRa), and reverse transcription of 1 µg of RNA was conducted with the cDNA synthesis kit (#R212, Vazyme) according to the manufacturer’s instructions. CHIKV (−) RNA was quantified as previously described (26). Briefly, cDNA was generated from 1 µg total RNA using the primer, 5′-GGCAGTATCGTGAATTCGATGCCGCTGTACCGT CCCCATTCC-3′, and the moloney murine leukemia virus (M-MLV) reverse transcriptase following the manufacturer’s instructions (#28025013, Invitrogen). After reverse transcription, the cDNA products were subjected to real-time PCR analysis to measure mRNA expression levels of tested genes. The threshold cycle for the indicated genes was normalized to that of the housekeeping gene *GAPDH* and shown as the relative mRNA level. Primer sequences for qPCR are shown in the supplemental information (Table S2).

### Plaque assay

After virus infection or viral RNA transfection in cells, the supernatants were used for plaque assays on monolayers of Vero cells seeded in 24-well plates. The cells were infected by incubation for 1 h at 37°C with serial dilutions of cell supernatants. After 1 h of infection, 2% methylcellulose was overlaid, and the plates were incubated for about 48 h. The overlay was removed, and cells were fixed with 4% paraformaldehyde for 15 min and stained with 1% crystal violet for 30 min before plaque counting.

### Virus binding and internalization assays

For virus binding assay, CHIKV-181/25 virions (MOI of 10) were added to control or YBX1-deficient HeLa cells in a 12-well plate and incubated on ice for 1 h. To remove unbound virions, cells were washed six times with ice-cold phosphate-buffered saline (PBS) and were harvested for RT-qPCR analysis. For internalization assay, following the on-ice incubation and washing, the cells were then incubated at 37°C for 1 h. Cells were then washed once with PBS and treated with 500 ng/mL proteinase K for 1 h on ice to stop endocytosis and degrade viruses that had not been internalized. After three additional washes with ice-cold PBS, cells were harvested for RT-qPCR analysis.

### RNA transfection and reporter assay

The control and YBX1-deficient HEK293T cells were transfected with 1 µg of *in vitro*-transcribed RNA using the Lipofectamine MessengerMax reagent (#LMRNA008, Invitrogen) according to the manufacturer’s instructions, and cells transfected with genomic viral RNA were cultured in the presence of 15 mM NH_4_Cl to prevent further rounds of infection. At the indicated times, cells were lysed with passive lysis buffer (#E1941, Promega). Luciferase assays were performed using the firefly or renilla luciferase assay kit (Promega).

### Immunofluorescence assay

The control and YBX1-deficient HeLa or HEK293T cells were infected with CHIKV-181/25 or CHIKV-181/25-nsP3-GFP, respectively, at an MOI of 50 for the indicated times. The cells were fixed with 4% paraformaldehyde for 30 min and permeabilized with 0.1% Triton X-100 in PBS for 10 min at 4°C. The cells were blocked with 1% bovine serum albumin in PBS and incubated with the indicated primary and secondary antibodies for 1 h. The nuclei were stained with 4′,6-diamidino-2-phenylindole. The stained cells were observed with a Leica confocal microscope (STELLARIS 8 WLL) under a ×63 oil objective.

### Transmission electron microscopy

The control and YBX1-deficient HEK293T cells were infected with CHIKV at an MOI of 50 for 24 h. Cells were scraped off with PBS and fixed with 2.5% glutaraldehyde overnight. After washing with 0.1 M phosphate buffer (pH 7.4) three times, cells were fixed with 1% osmium acid for 2 h and washed with 0.1 M phosphate buffer (pH 7.4) three times. Cells were subsequently fully dehydrated in a graded series of ethanol solutions and propylene oxide. An impregnation step was subsequently performed with a mixture of (2:1) acetone/epoxy resin and with a mixture of (1:1) acetone/epoxy resin and then left overnight in epoxy resin. Samples were embedded in epoxy resin, which was allowed to polymerize for 48 h at 60°C. Ultra-thin sections (80–100 nm) of these blocks were obtained with Leica EM UC7 Ultramicrotome. Sections were stained with 2% uranyl acetate and 5% lead citrate, and observations were made with a 120 kV transmission electron microscope (Talos L120C, Thermo Fisher).

### Co-immunoprecipitation assay

HEK293T cells were seeded on 10 cm dishes overnight. The cells were transfected with the indicated plasmids for 24 h. Cells were then lysed in l mL NP-40 lysis buffer (20 mM Tris-HCl, pH 7.5, 150 mM NaCl, 1 mM EDTA, 1% NP-40, 10 µg/mL aprotinin, 10 µg/mL leupeptin, and 1 mM phenylmethylsulfonyl fluoride). The lysates were centrifuged at 13,000 rpm for 10 min at 4°C. Supernatants were incubated with the indicated magnetic beads at 4°C overnight. Beads were washed six times with tris buffered saline with tween 20 (TBST). Samples were prepared and immunoblotted with the indicated antibodies. For input, 0.5% of whole-cell lysates were loaded on the gel.

### Pull-down assay

His-YBX1 and GST-CHIKV-nsP3 proteins were expressed in *E. coli* and purified by affinity chromatography. For protein pull-down assay, His-YBX1 (10 µg) and GST-CHIKV-nsP3 (10 µg) proteins were incubated with anti-His magnetic beads (15 µL) (#HY-K0209, MedChemExpress) at 4°C overnight. For RNA pull-down assay, CHIKV-181/25 genomic RNA was labeled with biotin by EZ-Link Psoralen-PEG3-Biotin (#29986, Thermo Fisher) as the manual described. Biotin-labeled RNA (10 µg), His-YBX1 (10 µg), and GST-CHIKV-nsP3 (10 µg) proteins were incubated with 10 µL streptavidin magnetic beads (#HY-K0208, MedChemExpress) at 4°C overnight. Beads were washed six times with TBST. Samples were prepared and immunoblotted with the indicated antibodies.

### Statistical analysis

Statistical analysis was performed with Prism version 8.0 (GraphPad). Statistical significance was analyzed by two-way analysis of variance followed by Dunnett’s test. Two-tailed unpaired (Student) *t*-test was performed if only two conditions were compared. Statistical significance was assigned when *P* values were <0.05. Error bars show mean and standard deviation (SD) (mean ± SD) unless otherwise specified. All data are representative of at least three independent experiments with similar results.

## Data Availability

All data are available in the article and supplemental material.

## SUPPLEMENTAL MATERIAL

Supplemental figures jvi.02015-24-s0001.pdf Figures S1 to S4.

Table S1 jvi.02015-24-s0002.xlsx siRNA sequences.

Table S2 jvi.02015-24-s0003.xlsx Primers used in RT-qPCR experiments.

ASM does not own the copyrights to Supplemental Material that may be linked to, or accessed through, an article. The authors have granted ASM a non-exclusive, world-wide license to publish the Supplemental Material files. Please contact the corresponding author directly for reuse.
