# Supplementary material for: YBX1 is required for assembly of viral replication complexes of chikungunya virus and replication of multiple alphaviruses
Source: J Virol. 2024 Dec 31;99(2):e02015-24. doi: 10.1128/jvi.02015-24 (PMC11852927; doi:10.1128/jvi.02015-24)
Supplement: Supplemental figures — Figures S1 to S4. [file jvi.02015-24-s0001.pdf]

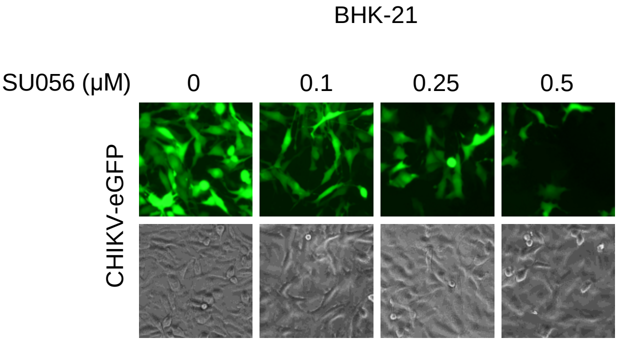

Fig. S1 SU056 inhibits CHIKV replication at a dose-dependent manner.

BHK-21 cells were pretreated with the indicated concentrations of SU056 for 12 h, and infected with CHIKV-eGFP (MOI=1) for 24 h before fluorescence microscopy.

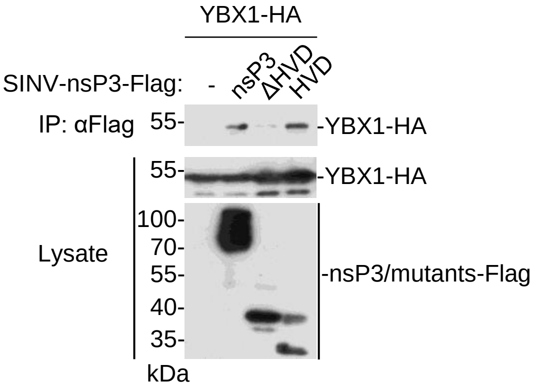

Fig. S2 YBX1 interacts with SINV nsP3 HVD.

YBX1 interacts SINV nsP3 HVD. HEK293T cells were transfected with indicated plasmids for 24 h. Co-immunoprecipitation and immunoblot analysis was performed with the indicated antibodies.

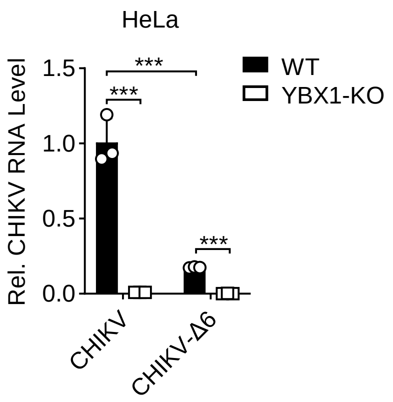

Fig. S3 YBX1 was required for replication of CHIKV-Δ6 virus

The control (Con) or YBX1-deficient (YBX1-KO) HeLa cells were infected with CHIKV or CHIKV-Δ6 at MOI of 1 for 12 h before RT-qPCR was performed. Data are normalized to the viral RNA level in the control cells. Data are represented as mean ± SD. ***P < 0.001.

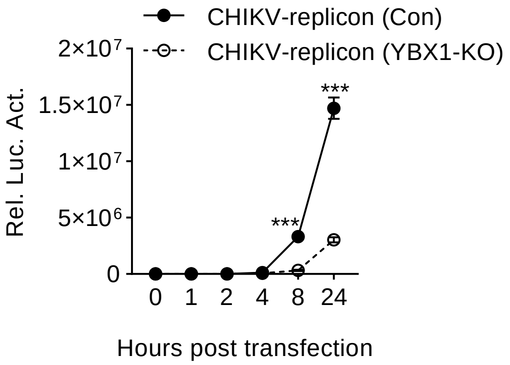

Fig. S4 YBX1 is essential for CHIKV RNA replication

The control (Con) or YBX1-deficient (YBX1-KO) HEK293T cells were transfected with in vitro transcribed CHIKV replicon RNA. The Rluc activity was assessed at the indicated times. Data are represented as mean ± SD. ***P < 0.001.
